# The cumulative prevalence of HIV-1 drug resistance in perinatal HIV

**DOI:** 10.1097/QAD.0000000000004202

**Published:** 2025-04-15

**Authors:** Jessica S. Glenn, Aisleen Bennett, Nicola Mackie, Hermione Lyall, Sarah Fidler, Graham Taylor, Caroline Foster

**Affiliations:** a Imperial College London; b Imperial College Healthcare NHS Trust; c Imperial College NIHR BRC Department of Infectious Disease, London, UK.

**Keywords:** antiretroviral therapy, drug resistance mutations, perinatal HIV

## Abstract

**Objective:**

To describe acquired drug resistance mutations (DRMs) among children and adults with perinatal HIV stratified by age.

**Design:**

A retrospective observational cohort study.

**Methods:**

Data on demographics, antiretroviral therapy (ART), viral load, CD4^+^ cell count, and lifetime cumulative acquired DRMs was collected and disaggregated by birth era; pre and post 2000; 0–24 and at least 25 years (*n* = 113 vs. 167).

**Results:**

Two hundred eighty individuals (median age 26 years, interquartile range 21–30), 235 (84%) Black ethnicity, 160 (57%) female, with median ART exposure 17 years. About 99.6% currently on ART, 205 (73%) integrase strand transfer inhibitor (INSTI) regimens, with 252 (90%) viral load less than 200 copies/ml. One hundred twenty-one of 280 (43%) acquired resistance to at least one ART class (37% 0–24 vs. 47% ≥ 25 years), 69/280 (25%) at least two (14 vs. 32%), and 13/280 (4.6%) at least three class; 11/13 (85%) aged at least 25 years. DRMs by ART class; 104/280 (37%), nonnucleoside reverse transcriptase inhibitor (NNRTI), 78 (28%) nucleoside reverse transcriptase inhibitor (NRTI), 15 (5%) protease inhibitor, and 4 (1%) INSTI. Uni/multivariate analysis; DRM acquisition was significantly associated with more than two anchor class exposure (*P* = 0.000), prior AIDS diagnosis (*P* = 0.001, 0.085), and early mono/dual NRTI exposure (*P* = 0.000, 0.029).

**Conclusion:**

Despite improved ART efficacy, DRMs limit treatment options, including long-acting injectable therapies with one-third having NNRTI-DRMs. Outcomes for second-generation INSTIs are promising with low rates of resistance but require continued monitoring. While multidrug resistance rates are lower in those born post2000, over one-third already have DRMs, highlighting the ongoing need for patient-centered approaches addressing adherence and novel ART class development.

## Introduction

Approximately 11 million children have acquired perinatal HIV (PaHIV) since 1990, with 2.58 million [confidence interval (CI) - 1.91–3.47] currently living with HIV [1,2]. Without access to antiretroviral therapy (ART) in low middle income countries (LMIC), 50% of infants will not survive to their second birthday, but early access to ART can result in near-normal life expectancy [1].

PaHIV cohorts are aging, with the majority in high-income countries (HICs) having transitioned to adult care and face the impacts of lifelong HIV and long-term ART exposure [3]. Earlier ART regimens relied on first-generation anchor drugs, including nonnucleoside reverse transcriptase inhibitors (NNRTIs) and unboosted protease inhibitors, with reduced efficacy, poor tolerability, and increased toxicity compared to current integrase strand transfer inhibitor (INSTI)-based regimens [4]. Initial rollout of triple therapy for children lagged behind that of adults, with reduced ART options and unpalatable formulations often resulting in inadequate dosing [5].

The introduction of second-generation INSTI, particularly dolutegravir (DTG)-based regimens, has markedly reduced NNRTI and protease inhibitor use [4]. The WHO recommended DTG-based therapy for both first-line and second-line ART in 2019, due to tolerance, potency, and high genetic barrier to resistance for all populations from 4 weeks of age [6]. However, adherence to daily oral regimens remain a challenge, increasing the risk of virological failure and the subsequent acquisition of drug resistance mutations (DRMs) [7]. In children, the risk of virological failure is higher due to a reduced number of appropriate ART agents and formulations, poor palatability, under-dosing with growth, drug stock-outs, reliance on carers for administration, subsequently compounded by the adherence challenges faced during adolescence [8,9]. The Joint United Nations Programme for HIV/AIDS (UNAIDS) set the 95–95–95 targets for 2030 where 95% of those living with HIV are aware of their status, of whom 95% are accessing ART, with 95% of those achieving sustained viral suppression [10]. As of 2023, stark gaps remain for children with targets achieved in 66–57–48 for those under 15 years compared to 87–77–73 for all ages [10].

Prior to the transition to dolutegravir, NNRTI-based regimens were the mainstay of treatment in LMIC with limited access to PIs [11,12]. Additionally, restricted access to viral load and resistance testing and limited robust second-line options resulted in rates of NNRTI-DRMs among earlier PaHIV cohorts of 44–93% (Table 1) [8,13–26]. Globally, rates of protease resistance are approximately 4–9% (range 0–20%) with triple class resistance documented in 4–23% of those living with PaHIV (Table 1). Although second-generation INSTIs have a high genetic barrier to resistance, drug resistance is emerging, with rates seemingly higher in children compared to adults, associated with prior nucleoside reverse transcriptase inhibitors (NRTIs) resistance in LMIC settings [14,20,21,27–31].

**Table 1:** Frequency of drug resistance in perinatal HIV cohorts by country, age, and drug class [32].

Author, year	Country	No. of participants (*N*)	Median age (years)	Study design	No. of patients with VF (%)	No. of patients with resistance assays (%)	No. of patients with DRMs (%)	No. ART classes of resistance (%)	Comments
Low/middle-income countries									
Dow, 2019 [13]	Tanzania	280	17	Prospective cross-sectional study	103/280 (37%)	71/103 (69%)	M184V–50/71 (70%) NNRTI –>63/70 (>90%) PI–1/33 (3%)	Triple–20/70 (29%)	Overall triple-class resistance- 20/280 (7%)
Bahemana, 2024 [14]	Tanzania	785	1-19		117/785 (15%)	74/117 (63%)	NNRTI–53/74 (72%) NRTI–50/74 (68%) PI–1/74 (1%) INSTI–3/74 (4%)	Dual–47/74 (64%)	
Makatini, 2019 [15]	South Africa	236	8	Retrospective cohort study	NA	236/236 (100%)	PI–22/236 (9%)		
Naidoo, 2022 [16]	South Africa	8762	14	Secondary analyses from surveys	182/398 (46%)	106/182 (58%)	DRMs–33/106 (31%) NNRTI–18/33 (55%)	Dual–13/33 (39%)	
Rubio-Garrido, 2021 [17]	Democratic Republic of Congo	55	14	Retrospective cohort study	49/55 (89%)	55/55 (100%)	NRTI–30/55 (55%) NNRTI–36/55 (65%) PI–5/55 (9%)	Single–7/55 (13%) Dual–23/55 (42%) Triple–6/55 (11%) Quadruple–1/55 (2%)	
Fokam, 2021 [8]	Cameroon	270	15	Cross-sectional study	106/270 (39%)	95/106 (90%)	NRTI–75/95 (79%) NNRTI–85/95 (89%) PI–4/95 (4%)	Dual–73/92 (79%) Triple–4/92 (4%)	Overall dual-class resistance–73/270 (27%) Overall triple-class resistance–4/270 (1%)
Djiyou Djeuda, 2024 [18]	Cameroon	280	10-19		157/280 (56%)	133/157 (85%)	DRMs–105/133 (79%) NNRTI–88/133 (66%) NRTI–74/133 (56%) PI–7/133 (5%) INSTI–26/133 (20%)		
Nyandiko, 2023 [19]	Kenya	480	8	Observational cohort study	167/480 (35%)	128/167 (77%)	DRMs–119/128 (93%) NRTI–114/128 (89%) NNRTI–119/128 (93%)	Dual–114/128 (89%)	Overall dual-class resistance–114/480 (24%)
Bello, 2024 [20]	Malawi	302	2-14	Cross-sectional survey	133/302 (44%)	INSTI–133/133 (100%) RT–131/133 (98%)	NRTI–56/133 (42%) NNRTI–88/133 (66%) PI–7/133 (5%) INSTI–22/133 (17%)		
Simon, 2024 [21]	Malawi	302	10		302/302 (100%)	117/302 (39%)	INSTI–31/117 (26%)	Single–33/117 (28%) Dual–18/117 (15%) Quadruple–2/117 (2%)	
Taveria, 2024 [22]	Mozambique	125	Infants	Randomised control trial	73/125 (58%)	73/125 (58%)	NRTI–23/73 (32%) NNRTI–39/73 (53%) PI–1/73 (1%) INSTI–2/73 (3%)		
Kityo, 2024 [23]	Africa	908	Children	Randomised control trial	124/908 (14%)	RT/PR–86/124 (69%) INSTI–79/124 (64%)	NNRTI–82/86 (95%) NRTI–53/86 (62%) PR–1/86 (1%) INSTI–2/79 (3%)		
Dahourou, 2024 [24]	West Africa	449	14	Stepped-wedge trial	167/439 (38%)	12/167 (7%)	NRTI–7/12 (58%) INSTI–3/12 (25%)		
Etima, 2024 [25]	North Central Uganda	27	10.7		27/27 (100%)	RT/PR–27/27 (100%) PR–25/27 (93%) INSTI–22/27 (81%)	NNRTI–11/25 (44%) PR–1/5 (20%) INSTI–5/22 (23%)		
Perry, 2024 [26]	Eswatini	25	2.7	Retrospective review	25/25 (100%)	25/25 (100%)	NRTI–4/25 (16%) NNRTI–11/25 (44%) PI–0/25 INSTI–0/25		
High-income countries									
Van Dyke, 2016 [32]	United States of America	234	15	Prospective cohort study	234/234 (100%)	234/234 (100%) INSTI–11/234 (5%)	DRMs–175/234 (75%) NRTI–142/234 (61%) NNRTI–105/234 (45%) PI–80/234 (34%) INSTI–3/11 (27%)	Single–70/234 (30%) Dual–63/234 (27%) Triple–43/234 (18%)	
Collins, 2017 [33]	United Kingdom	1907	17	Prospective cohort study	357/481 (74%)	RT, PR–381/644 (59%) INSTI–0/5	NRTI–186/291 (64%) NNRTI–198/291 (68%) PI–55/291 (19%) INSTI–0/5	Single–76/291 (26%) Dual–128/291 (44%) Triple–34/291 (12%)	
Rojas, 2018 [34]	Spain	245	9	Retrospective study	200/218 (92%)	RT–172/200 (86%) PR–168/200 (84%)	DRMs–139/190 (73%) NRTI–111/172 (65%) NNRTI–62/172 (36%) PI–59/168 (35%)		
Ungaro, 2019 [35]	Italy	94	18+	Retrospective cohort study	NA	94/94 (100%)	DRMs–74/94 (79%) NRTI–74/94 (79%) NNRTI–61/94 (65%) PI–33/94 (35%) INSTI–7/94 (7%)	Dual–39/94 (41%) Triple–19/94 (20%) Quadruple–6/94 (6%)	

A PubMed Search was conducted for perinatal HIV and drug resistance mutations, with studies from 2017 onwards included. Studies are arranged in chronological order and by country in two categories: high-income countries and low-income and middle-income countries. ART, antiretroviral therapy; DRMs, drug-resistance mutations; INSTI, integrase strand transfer inhibitors; NA, not applicable; NNRTI, nonnucleoside reverse transcriptase inhibitors; NRTI, nucleoside reverse transcriptase inhibitor; PI, protease inhibitors; PR, protease; RT, reverse transcriptase; VF, virological failure.

In HIC, a national prospective UK cohort study (registered for care 1996–2014) followed 1907 children through to adult care (median age 17 years) [33]. Of 291 with resistance data, 26% had single, 44% dual and 12% triple class resistance. NNRTI resistance was most common (68%), followed by NRTIs (64%) with PIs at 19%. Comparable rates are reported in other PaHIV cohorts in the United States of America and Europe, although data are surprisingly limited (Table 1) [32,34,35]. Individuals living with PaHIV currently face a lifetime on ART with the potential for onward transmission of resistant variants to partners and offspring for those that struggle with adherence [36]. Current long-acting ART regimens require no prior failure on NNRTI or INSTI regimens, limiting their use for treatment experienced PaHIV cohorts [37].

This study aims to describe the lifetime prevalence and risk factors associated with acquired HIV-1 DRMs in a cohort with PaHIV and discusses the impact on treatment options.

## Materials and methods

### Design

A retrospective, observational, single-center cohort study of children, adolescents, and adults living with PaHIV (CAPaHIV) registered for National Health Service (NHS) care at a tertiary HIV service in London between January 1, 2023, and February 1, 2024. Children with PaHIV are seen in the Family Clinic service and transition to specialist PaHIV service in adult care for lifelong follow-up as previously described [38].

Data was extracted from electronic and paper patient records, Virtual Clinic and virology databases from care entry until April 1, 2024.

### Outcome definitions

Patients’ ages were categorized as of December 31, 2024, by WHO age bands: 0–14, 15–19, 20–24, 25–29, and 30+ years. Sub-analysis was also performed with age groups combined 0–24 years and 25 years and above, reflecting birth prior to and after the introduction of combination therapy in HIC [39]. Ethnicities were grouped according to the UK government standard categories: Asian, Black African, Mixed, White, and Other [40]. Birthplaces were classified as UK or abroad. Sex was defined as biological sex at birth.

HIV-1 viral subtypes were obtained from past genotypic resistance assays where available. Routine genotyping in NHS care began around 2000, so resistance assays were unavailable for individuals on ART with sustained viral suppression from before 2000, and for those who transferred into the service with suppressed viral loads and without subsequent virological failure [41]. The frequency of genotyping followed national guidance and occurred at baseline for those initiating ART after the year 2000, at the time of each new episode of virological failure and for those with persistent viraemia on ART at 6 to 12 monthly intervals, The lifetime cumulative major mutations from all sequences available for each patient were recorded.

Prior AIDS diagnoses were confirmed using The Centers for Disease Control and Prevention (CDC) criteria, adapted accordingly for children and adults [42,43]. Individuals were assigned a CDC-C status if they had an AIDS-defining illness, or CD4^+^ cell count thresholds less than 200 cells/μl or less than 15% above 5 years of age, less than 500 cells/μl aged 1–5 years, and CD4^+^ cell count less than 750 cells/μl in the first year of life [44,45].

Co-infection with hepatitis B or past hepatitis C was recorded [46].

Latest clinical outcomes included absolute CD4^+^ cell count, CD4:CD8 ratio, and viral load from their most recent routine blood draw, dated at earliest January 1, 2023.

Viral loads were banded accordingly: <50 copies/ml (undetectable), 50–199 copies/ml (low-level viraemia), 200–999 copies/ml (low-level virological failure), and at least 1000 copies/ml (virological failure) [47,48].

Pretreatment factors such as nadir (lowest historical) CD4^+^ cell count were recorded where available [49]. Nadir CD4^+^ cell counts were banded accordingly: less than 200 cells/μl/<15% (AIDS diagnosis see age related criteria above), 200–349 cells/μl (late HIV diagnosis), and at least 350 cells/μl [50–52].

For treatment history, current and past ART regimens and their duration were recorded. Total years since ART initiation and anchor class exposure (the number of anchor drugs prescribed throughout each patient's lifetime) were calculated. Anchor drugs were defined as NNRTI, protease inhibitor, and INSTI drug classes typically paired with a dual NRTI back bone [53]. Historical suboptimal mono/dual NRTI exposure prior to effective triple regimens were recorded, including the duration of exposure.

Major DRMs were extracted from genotypic sequencing reports and categorized by drug class, according to the Stanford HIV Drug Resistance Database (https://hivdb.stanford.edu/hivdb/by-mutations/). Polymorphisms and minor mutations were not included. Baseline DRMs (transmitted DRMs) predating ART initiation were excluded from the analysis [7]. All sequences were performed on HIV RNA at the time of viraemia by Sanger sequencing until 2017 and subsequently by next-generation sequencing with the addition of integrase to standard sequencing of protease and reverse transcriptase from 2008. No analyses of proviral DNA were included. This project focuses on cumulative acquired resistance over the lifetime of the individual, so both archived and current mutations were noted. NRTI mutations were grouped into M184 V/I, thymidine analogue mutations (TAMs), and other resistance-conferring mutations [54]. The number of resistance classes was recorded, with multiclass resistance defined as resistance to two or more ART classes [55].

### Statistical analysis

The data was anonymized, summarizing categorical variables with numbers and percentages. Distributions of continuous variables were examined and categorical variables tabulated. Two-sided *t*-tests compared independent means for data with normal distribution. For nonnormally distributed continuous variables, medians and interquartile ranges (IQRs) were reported.

Chi-square or Fisher's exact tests (depending on the number of variables) compared categorical variables between groups, with significance set at a *P* value less than 0.05. For variables with more than two groups, comparisons were made to the baseline group.

Risk factors for development of DRMs were identified using a logistic regression model, with the outcome variable the presence or absence of DRMs. The model was built using a forward stepwise approach (Supplementary materials page 8, supplementary table 4). Variables were included in multivariable analysis based on *a priori* knowledge of potential associations with DRM. The following variables were included: age in years, ethnicity, sex, country of birth, weight, height, pretreatment viral load, pretreatment CD4^+^ cell count, anchor class exposure (number), hepatitis B infection, hepatitis C infection, prior Centers for Disease Control and Prevention C status (CDC-C) diagnosis, years since ART initiation, prior mono/dual NRTI therapy exposure, HIV subtype clade.

All variables with a *P* value of less than 0.1 on univariable analysis were tested for inclusion in the final model. Nested models were compared using likelihood ratio tests. All variables with a *P* value less than 0.1 during this process were retained in the final model. Within this model, significance was set at *P* value less than 0.05. Age was included in the model *a priori*. Anchor class exposure was categorized into 2 or less or more than 2 for the multivariate model. Additional sensitivity analysis was undertaken to explore the effect of exposure to different drug classes and age cohort on the results (supplementary materials page 9, supplementary tables 5–12). Analyses were conducted using Stata 13 software (StataCorp. 2013. Stata Statistical Software: Release 13, StataCorp LLC College Station, Texas, USA).

### Ethical considerations

The study was registered as a service evaluation with the Imperial College Health Trust (ICHT) audit office (registration number PAED_60) and was approved in the adult ICHT HIV/GUM research meeting. As only anonymized routinely collected clinical data were used within a password-protected anonymized database, research ethical approval was not required under UK Health Research Authority (HRA) guidelines [56].

## Results

Of the 280 individuals living with PaHIV registered for care, 217 (78%) had sequences available from any time point, 121 (56%) of whom had acquired DRM in their lifetime to date. The current median age of the cohort was 26 years (IQR 21,30, range 2–40) and 160/280 (57%) were female at birth (Table 2). Individuals were predominantly of Black ethnicity (235/280, 84%) with 162/280 (58%) born outside the UK, and 152/280 (54%) having a prior CDC-C diagnosis.

**Table 2:** Demographic data, clinical data, and drug resistance mutations.

		Age group (years)						
		0–14	15–19	20–24	25–29	30+	0–24	25+
	*n* (%)	24 (9)	34 (12)	55 (20)	88 (31)	79 (28)	113 (40)	167 (60)
Demographic data								
Age	Median (IQR)	12 (6,13)	17 (16,18)	22 (21,23)	27 (25,28)	32 (31,34)	19 (15,22)	29 (27,32)
Female	*n* (%)	14 (58)	15 (44)	33 (60)	54 (61)	44 (56)	62 (55)	98 (59)
Ethnicity								
Black African	*n* (%)	16 (67)	27 (79)	47 (85)	77 (88)	68 (86)	90 (80)	145 (87)
Asian	*n* (%)	0	0	3 (5)	1 (1)	2 (3)	3 (3)	3 (2)
Mixed	*n* (%)	3 (13)	3 (9)	5 (9)	5 (6)	2 (3)	11 (10)	7 (4)
Caucasian	*n* (%)	4 (17)	4 (12)	0	4 (5)	7 (9)	8 (7)	11 (7)
Other	*n* (%)	1 (4)	0	0	1 (1)	0	1 (1)	1 (1)
Country of birth								
Abroad	*n* (%)	15 (63)	13 (38)	26 (47)	54 (61)	54 (68)	54 (48)	108 (65)^e^
UK	*n* (%)	9 (38)	21 (62)	29 (53)	34 (39)	25 (32)	59 (52)	59 (35)
Clade								
Resistance sequences available	*n* (%)	18 (75)	21 (62)	44 (80)	70 (80)	64 (81)	83 (74)	134 (80)
C	*n* (%)	9 (50)	12 (57)	21 (48)	29 (41)	16 (25)	42 (51)	45 (34)^a^
B	*n* (%)	2 (11)	1 (5)	2 (5)	8 (11)	5 (8)	5 (6)	13 (10)
Other^a^	*n* (%)	7 (39)	8 (38)	21 (48)	33 (47)	43 (67)	36 (43)	76 (57)
Hepatitis co-infection								
Hepatitis B	*n* (%)	0	0	0	2 (2)	5 (6)	0	7 (4)
Hepatitis C (past)	*n* (%)	0	0	1 (2)	0	1 (1)	1 (1)	1 (1)

Prior CDC-C status	*n* (%)	11 (46)	13 (38)	28 (51)	50 (57)	48 (61)	54 (48)	98 (59)
Clinical data								
Latest clinical data								
Absolute CD4^+^ (cells/μl)	Median (IQR)	584 (660,1092)	730 (585,918)	675 (470,833)	628 (456, 831)	601 (419,813)	730 (566,954)	615 (437,829)
CD4:CD8	Median (IQR)	1.3 (0.8,1.6)	1.3 (1.0,1.6)	1.0 (0.6, 1.5)	0.9 (0.6, 1.2)	0.9 (0.6,1.3)	1.1 (0.7,1.6)	0.9 (0.6,1.3)
VL (<50 c/ml)	*n* (%)	20 (83)	30 (88)	49 (89)	70 (80)	67 (85)	99 (88)	137 (82)
VL(50–199 c/ml)	*n* (%)	3 (13)	0	3 (5)	6 (7)	4 (5)	6 (5)	10 (6)
VL (200–999 c/ml)	*n* (%)	1 (9)	2 (6)	2 (4)	1 (1)	3 (4)	5 (4)	4 (2)
VL (≥1000 c/ml)	*n* (%)	0	2 (6)	1 (2)	11 (13)	5 (6)	3 (3)	16 (10)
Nadir CD4^+^ cell count								
Available	*n* (%)	24 (100)	34 (100)	51 (93)	73 (83)	62 (78)	109 (96)	135 (81)
<200 cells/μl	*n* (%)	3 (13)	7 (21)	11 (22)	33 (45)	35 (56)	21 (19)	68 (50)
200–349 cells/μl	*n* (%)	2 (8)	3 (9)	12 (24)	25 (34)	20 (32)	17 (16)	45 (33)
≥350 cells/μl	*n* (%)	19 (79)	24 (71)	28 (55)	15 (21)	7 (11)	71 (65)	22 (16)^a^
ART history								
Years since ART initiation	Median (IQR)	5 (2,11)	14 (8,16)	15 (9,20)	20 (15,24)	20 (16,26)	11 (6,17)	20 (16,24)
Past ART exposure								
INSTI	*n* (%)	23 (96)	31 (91)	46 (84)	74 (84)	67 (85)	100 (89)	141 (84)
NNRTI	*n* (%)	11 (46)	24 (71)	42 (76)	74 (84)	68 (86)	77 (68)	142 (85)^e^
bPI	*n* (%)	10 (42)	19 (56)	33 (60)	68 (77)	61 (77)	62 (55)	129 (77)^e^
Unboosted PI	*n* (%)	0	0	3 (5)	12 (14)	18 (23)	3 (3)	30 (18)^e^
Other^b^	*n* (%)	0	2 (6)	0	7 (8)	4 (5)	2 (2)	11 (7)
Mono/dual NRTI therapy	*n* (%)	0	0	1 (2)	8 (9)	20 (25)	1 (1)	28 (17)^e^
Anchor class exposure	Median (IQR)	2 (1,2)	2 (2,3)	2 (2,3)	3 (2,3)	3 (2,3)	2 (2,3)	3 (2,3)
Current anchor class								
INSTI	*n* (%)	23 (96)	28 (82)	44 (80)	59 (67)	51 (65)	95 (84)	110 (66)^e^
NNRTI	*n* (%)	1 (4)	2 (6)	6 (11)	7 (8)	7 (9)	9 (8)	14 (8)
bPI	*n* (%)	0	4 (12)	5 (9)	15 (17)	16 (20)	9 (8)	31 (19)^e^
bPI+ INSTI	*n* (%)	0	0	1 (2)	5 (6)	4 (5)	1 (1)	9 (5)
NNRTI + INSTI^c^	*n* (%)	0	0	0	2 (2)	1 (1)	0	3 (2)
NIL^d^	*n* (%)	0	0	1 (2)	0	0	1 (1)	0
Resistance data								
Number of classes of resistance								
0	*n* (%)	15 (63)	26 (76)	30 (55)	47 (53)	41 (52)	71 (63)	88 (53)
1	*n* (%)	7 (29)	4 (12)	15 (27)	16 (18)	10 (13)	26 (23)	26 (16)
2	*n* (%)	2 (8)	4 (12)	8 (15)	22 (25)	20 (25)	14 (12)	42 (25)^e^
3	*n* (%)	0	0	2 (4)	3 (3)	6 (8)	2 (2)	9 (5)
4	*n* (%)	0	0	0	0	2 (3)	0	2 (1)
Multi-Class (2+)	*n* (%)	2 (8)	4 (12)	10 (18)	25 (28)	28 (35)	16 (14)	53 (32)^e^
NRTI								
Any NRTI	*n* (%)	6 (25)	4 (12)	12 (22)	27 (31)	29 (37)	22 (20)	56 (34)^e^
DRM/person	Median (IQR)	1 (1,2)	1 (1,2)	1 (1,2)	3 (2,4)	4 (1,5)	1 (1,2)	3 (1.,5)
M184V/I	*n* (%)	6 (25)	3 (9)	12 (22)	19 (22)	20 (25)	21 (19)	39 (23)
TAMs	*n* (%)	1 (4)	1 (3)	4 (7)	2 (2)	4 (5)	6 (5)	6 (4)
Other	*n* (%)	1 (4)	1 (3)	3 (5)	13 (15)	14 (18)	5 (4)	27 (16)^e^
NNRTI								
Any NNRTI	*n* (%)	5 (21)	7 (21)	22 (40)	35 (40)	35 (44)	34 (30)	70 (42)
DRM/person	Median (IQR)	1 (1,1)	1 (1,2)	2 (1,2)	2 (1,2)	2 (1,2)	1 (1,2)	2 (1,2)
PI								
Any PI	*n* (%)	0	0	2 (4)	6 (7)	7 (9)	2 (2)	13 (8)^e^
DRM/person	Median (IQR)	NA	NA	2 (1,2)	1 (1, 2)	1 (1,2)	2 (1,2)	1 (1,2)
INSTI								
Any INSTI	*n* (%)	0	1 (3)	1 (2)	1 (1)	1 (1)	2 (2)	2 (1)

ART, antiretroviral therapy; bPI, boosted protease inhibitor; CDC-C, The Centers for Disease Control and Prevention Criteria C status; INSTI, integrase strand transfer inhibitor; IQR, interquartile range; NNRTI, nonnucleoside reverse transcriptase inhibitor; NRTI, nucleoside reverse transcriptase inhibitor; PI, protease inhibitor; UK, United Kingdom; VL, viral load.

a Other clades included A, D, F, G, AE, AG, AD, RKK, FK, DF, GJ.

b Other ART refers to enfuvirtide (T20) and maraviroc (MRV).

c Long-acting injectable cabotegravir and rilpivirine.

d One patient has thus far declined ART; elite controller:VL <50 copies/ml, CD4^+^ 1116 cells/μl, CD4 : 8 1.6.

e When there was a significant difference between the two groups (0-24 and 25+). *P* < 0.05 was considered significant. For further detail on statistical tests used, please refer to Supplementary Table 2.

At last follow-up, 252/280 (90%) had a viral load of less than 200 copies/ml with 236/280 (84%) less than 50 copies/ml with a median CD4^+^ cell count of 685 (IQR 463, 861) cells/μl (Table 2). All bar one 20-year old individual [an elite controller, viral load persistently <50 c/ml, CD4^+^ cell count 1116 (44%), CD4 : 8 1.6, with no DRMs on baseline sequencing who continues to decline ART] 279 (99.6%) were currently on ART; the majority 205 (73%) on INSTI-based regimens (Table 2). Of those with a nadir CD4^+^ cell count available, 151/244 (62%) were less than 350 cells/μl. Median duration since ART initiation was 17 (IQR 11,22) years, with a median exposure of 2 (IQR 2,3) anchor classes, with 241/280 (86%) ever exposed to INSTIs, 219 (78%) to NNRTIs, 191 (68%) to boosted protease inhibitors, and 33 (12%) to unboosted protease inhibitors (Table 2). Thirteen individuals (5%) had past exposure to other ART classes including entry inhibitors: maraviroc and enfurvirtide with 29 (10%) having had mono/dual NRTI exposure prior to triple therapy.

Of the 217/280 (78%) with genotypic testing for resistance available, 87 (40%) had subtype C virus, 18 (8%) subtype B, 63/280 (23%) subtype undocumented, with 121/217 (56%) having acquired DRM (Table 2). Of the 159 (57%) with no evidence of resistance, 96/159 (60%) had wild-type virus sequenced and 63/159 (40%) had sustained virological suppression and no sequence available. Two younger individuals (<10 years) had transmitted drug resistance to NNRTIs on baseline sequencing but experienced no subsequent virological failure on INSTI-based regimens. In the cohort, overall 121/280 (43%) had documented evidence of any acquired drug resistance mutations with 52/280 (19%) having single class resistance, 56 (20%) dual, 11 (4%) triple, and two (1%) quadruple (Table 2). NNRTI resistance mutations were seen in 104/280 (37%), NRTI in 78/280 (28%), protease in 15/280 (5%), and integrase mutations in 4/280 (1%) (Table 2). Integrase resistance occurred in four individuals; two treatment-experienced young adults with persistent viremia on darunavir/cobisistat/emtricitabine/tenofovir alafenamide with dolutegravir (1) and bictegravir/emtricitabine/tenofovir alafenamide (1). A third young adult with prior NNRTI-failure (K103N alone) unable to tolerate oral ART, CD4^+^ cell count less than 100 cells/μl, switched to LA-cabotegravir/rilpivirine, experienced viral rebound with extensive dual class resistance 2 years later despite never missing an injection, with the case previously described [57]. The final adolescent experienced viral rebound after 11 years suppressed on first-line therapy with DTG, abacavir, and lamivudine associated with a period of poor adherence with details recently published [29].

Univariate analysis identified several risk factors associated with the acquisition of DRMs: older age (*P* = 0.052), female sex at birth (*P* = 0.082), prior CDC-C diagnosis (*P* = 0.001), pretreatment viral load more than 100 000 (*P* = 0.016), >2 anchor drug class exposure (*P* = 0.000), years since ART initiation (*P* = 0.000), and prior mono/dual NRTI therapy exposure (*P* = 0.000) (Table 3). By multivariate analysis, more than 2 anchor class exposure (*P* = 0.000) and prior mono/dual NRTI therapy (*P* = 0.029) were retained as significant risk factors.

**Table 3:** Univariate and multivariate analysis of factors associated with presence of drug resistance mutations.

	DRM present *n* (%)	DRM absent *n* (%)	Univariate analysis OR (95% CI), *P*	Multivariate analysis (*n* 280) OR (95% CI), *P*
Age in years (280)				
0–14	9 (38)	15 (62)		
15–19	8 (24)	26 (76)		
20–24	25 (45)	30 (55)		
25–29	41 (47)	47 (53)		
30+	38 (48)	41 (52)	1.21 (1.00, 1.46), 0.052	0.89 (0.69, 1.15) 0.374
Sex at birth (280)				
Male	59 (49)	61 (51)		
Female	62 (39)	98 (61)	0.65 (0.40, 1.06), 0.082	
Country of birth (272)				
UK	57 (49)	61 (51)		
Abroad	59 (38)	95 (62)	0.66 (0.41, 1.08), 0.100	
Pretreatment VL (162)				
> 100,000	43 (49)	44 (51)		
< 100, 000	23 (31)	52 (69)	2.21 (1.14, 4.28), **0.016**	
Pretreatment absolute CD4^+^ cells/μl (169)				
0–200	20 (47)	23 (53)		
200–350	12 (40)	18 (60)		
>350	37 (39)	59 (61)	0.86 (0.60, 1.23), 0.395	
HIV subtype Clade				
B	12 (66)	6 (33)		
C	42 (48)	45 (52)		
Other	67 (38)	108 (62)	1.25 (0.97, 1.62) 0.087	
Anchor class exposure (280)				
≤ 2	29 (20)	113 (80)		
> 2	92 (67)	46 (33)	7.79 (4.2, 14.37), **0.000**	6.35 (3.60, 11.19), **0.000**
Prior CDC-C diagnosis (280)				
No	41 (32)	87 (68)		
Yes	80 (53)	72 (47)	2.36 (1.43, 3.89), **0.001**	1.65 (0.93, 2.90) 0.085
Years since ART initiation (280)				
0–9	13 (23)	44 (77)		
10–19	48 (41)	70 (59)		
20+	60 (57)	45 (43)	2.00 (1.45, 2.75), **0.000**	1.41 (0.90–2.21), 0.132
Prior mono/dual NRTI therapy exposure (280)				
No	100 (39)	154 (61)		
Yes	21 (81)	5 (19)	6.47 (2.29, 18.30), **0.000**	3.51 (1.14, 10.91), **0.029**
Ethnicity				
Black African	101 (43)	134 (57)		
Asian	3 (50)	3 (50)		
Mixed	5 (28)	13 (72)		
Caucasian	11 (58)	8 (42)		
Other	1 (50)	1 (50)	1.13 (0.84, 1.51) 0.42	

*P* < 0.1 was considered significant. All significant *P* values are in bold. ART, antiretroviral therapy; CDC-C, The Centers for Disease Control and Prevention C status; DRM, drug resistance mutation; OR, odds ratio; UK, United Kingdom; VL, viral load.

When comparing between age groups, all data are presented as those born after 2000 (age <25 years) vs. those born before 2000 (age 25–40 years). The younger cohort were more likely to be UK born (52 vs. 35%; *P* = 0.007), have started ART with a CD4^+^ cell count at least 350 cells/μl (65 vs. 16%; *P* < 0.0001), and have subtype C virus (51 vs. 34%; *P* = 0.016). While current viral suppression (<200 c/ml) was high in both groups (93 vs. 88%; *P* = 0.225), a higher proportion of the older age group had current virological failure more than 1000 c/ml (3 vs. 10%; *P* = 0.028). Past exposure to mono/dual NRTI therapy (1 vs. 17%; *P* < 0.0001) and unboosted protease inhibitors (3 vs. 18%; *P* < 0.0001) was almost entirely limited to those born before 2000. INSTI exposure was high in both groups (89 vs. 84%; *P* = 0.382) with NNRTI (68 vs. 85%; *P* = 0.001) and boosted protease inhibitor exposure (55 vs. 77%; *P* = 0.0001) more frequent in the older cohort. The risk of acquisition of multidrug (2+ class) resistance was higher in the older age group (14 vs. 32%; *P* = 0.001) with higher rates of NRTI resistance (20 vs. 34%; *P* = 0.01) and NNRTI-DRMs (30 vs. 42%; *P* = 0.058) (Supplementary Table 2).

## Discussion

In this cohort of 280 individuals born with perinatal HIV, current median age 26 years, 43% had acquired HIV-1 drug resistance mutations during an average 17 years of antiretroviral therapy. Reassuringly, rates of triple+ class resistance (NRTI+NNRTI+PI 3.9%, NRTI+NNRTI+PI+INSTI 0.7%) were less than 5%, with all receiving licensed ART regimens (supplementary table 3). The acquisition of DRMs was associated with total years since ART initiation, and prior mono/dual NRTI exposure before triple combination therapy became available for children in the late 1990 s. However, it is of concern that even in those born in the era of effective combination therapy, more than one-third (37%) had acquired DRMs, with 14% having resistance to two or more classes, as they transition to adult care and includes two young adults with integrase resistance.

The current immunological status of the cohort is good with a median CD4^+^ cell count 685 cells/μl and the majority (90%) are virally suppressed (<200 copies/ml) [58]. While this falls short of the UNAIDS’ goals of 95% accessing ART being virally suppressed, it is higher than many comparable PaHIV youth cohorts, where suppression rates range from 44 to 88% and are typically lower than in adult cohorts in the same setting [1,8,13–26,32–35]. It is notable that less than one quarter (63/280; 22.5%) had no genotypic sequences available due to sustained viral suppression either since 2000 or entered the UK on suppressive ART [28,29,33–35,37,57].

Forty-three percent had resistance to at least one ART class (NNRTI 37%, NRTI 22%, protease inhibitor 5%, INSTI 1.4%), with a quarter having dual+ and 4.6% triple+ class resistance (Fig. 1   a). Rates of triple-class resistance are lower than reported in other perinatal cohorts from Europe and the United States (3.9 vs. 12–20%), despite this cohort being almost a decade older (median age 26 years vs. 9–18+ years) [32–35]. When comparing rates of DRMs with perinatal cohorts in LMIC settings, rates of multiclass resistance appear similar, although the LMIC cohorts are significantly younger, and the classes of ART exposure more limited (Table 1) [8,13,16,18,19,21]. In an adolescent population in the Cameroon (*n* = 270, median age 15), 27% had dual-class resistance, similar to 24% in a younger Kenyan cohort (*n* = 480, median age 8) and comparable to 20% in this study [8,19]. Triple-class resistance was reported in 1% of the Cameroonian cohort but 7% in a Tanzanian cohort (*n* = 280, mean age 17 years) [8,13].

**Fig. 1:**
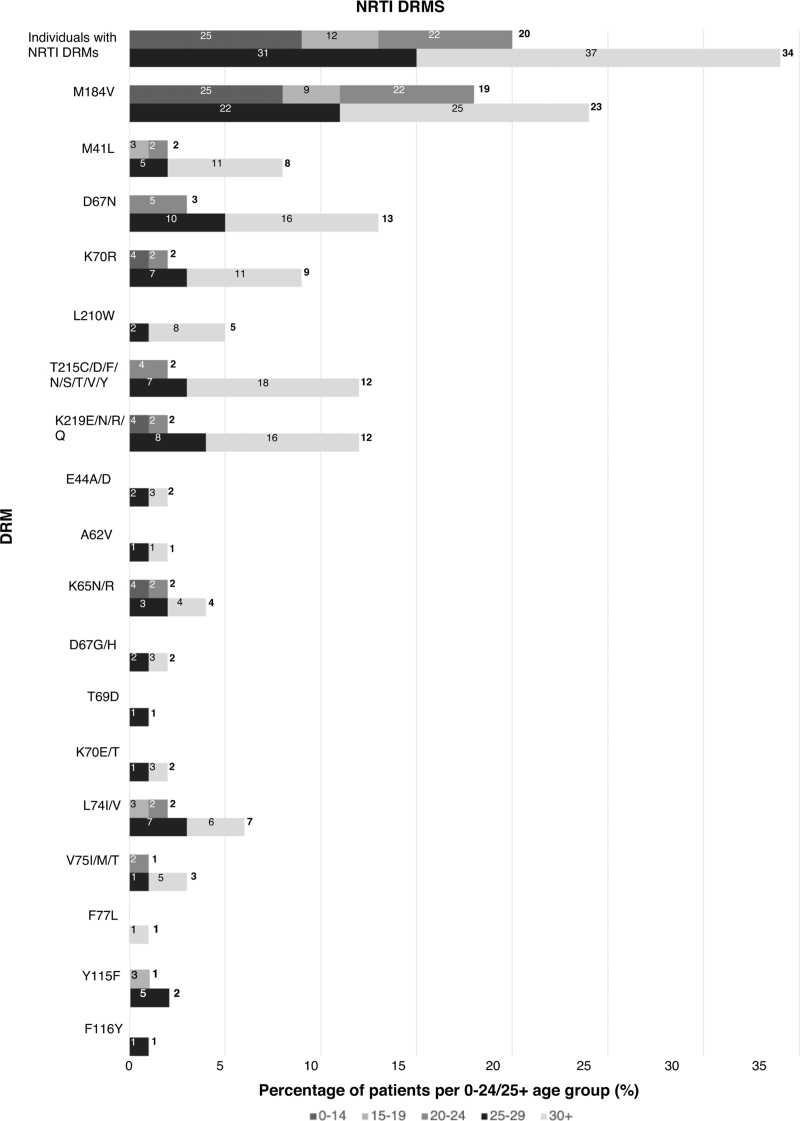
Distribution of age groups for number of resistance classes and antiretroviral therapy class-specific resistance.

The numbers within the bars correspond to the percentage of patients with the mutation per smaller age group (e.g., 0–14, etc.). The numbers outside the bar correspond to the percentage of patients in the larger age group (0–24, 25+) with the mutation. ART, antiretroviral therapy; INSTI, integrase strand transfer inhibitor; NNRTI, nonnucleoside reverse transcriptase inhibitor; NRTI, nucleoside reverse transcriptase inhibitor; PI, protease inhibitor.

**Fig. 1 (Continued):**
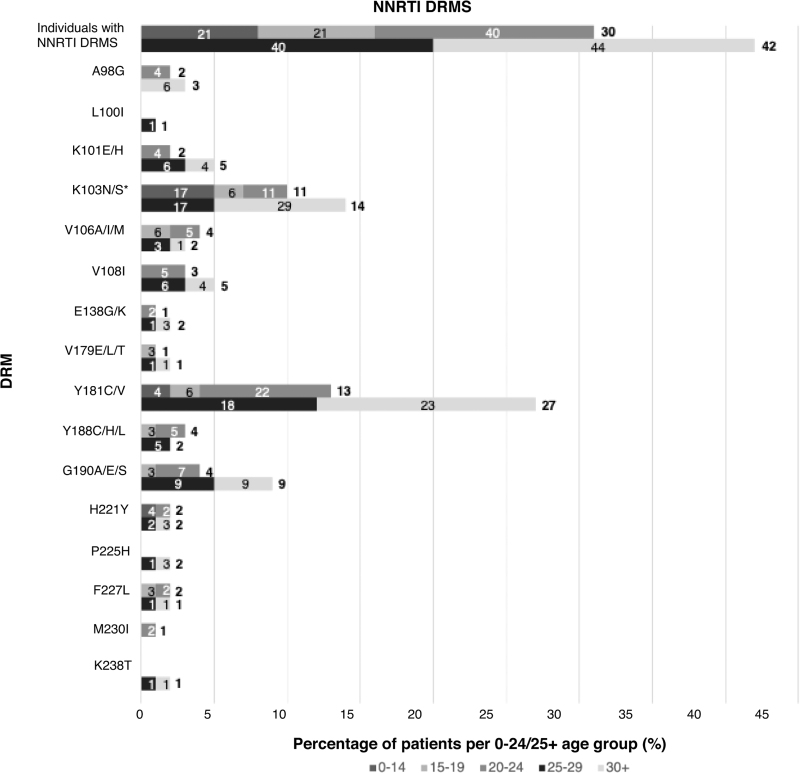
Distribution of age groups for number of resistance classes and antiretroviral therapy class-specific resistance.

The numbers within the bars correspond to the percentage of patients with the mutation per smaller age group (e.g., 0–14, etc.). The numbers outside the bar correspond to the percentage of patients in the larger age group (0–24, 25+) with the mutation. ART, antiretroviral therapy; INSTI, integrase strand transfer inhibitor; NNRTI, nonnucleoside reverse transcriptase inhibitor; NRTI, nucleoside reverse transcriptase inhibitor; PI, protease inhibitor.

**Fig. 1 (Continued):**
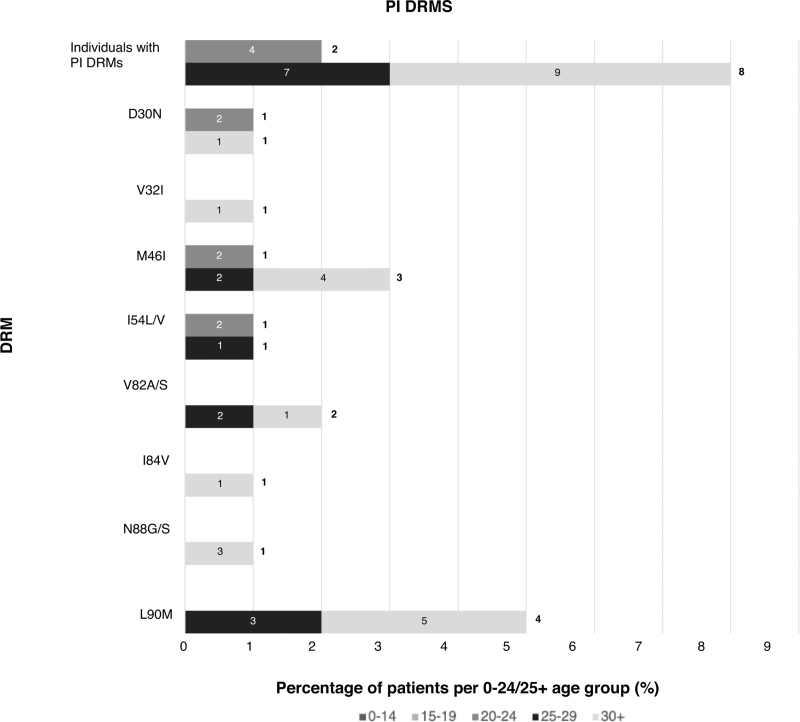
Distribution of age groups for number of resistance classes and antiretroviral therapy class-specific resistance.

The numbers within the bars correspond to the percentage of patients with the mutation per smaller age group (e.g., 0–14, etc.). The numbers outside the bar correspond to the percentage of patients in the larger age group (0–24, 25+) with the mutation. ART, antiretroviral therapy; INSTI, integrase strand transfer inhibitor; NNRTI, nonnucleoside reverse transcriptase inhibitor; NRTI, nucleoside reverse transcriptase inhibitor; PI, protease inhibitor.

**Fig. 1 (Continued):**
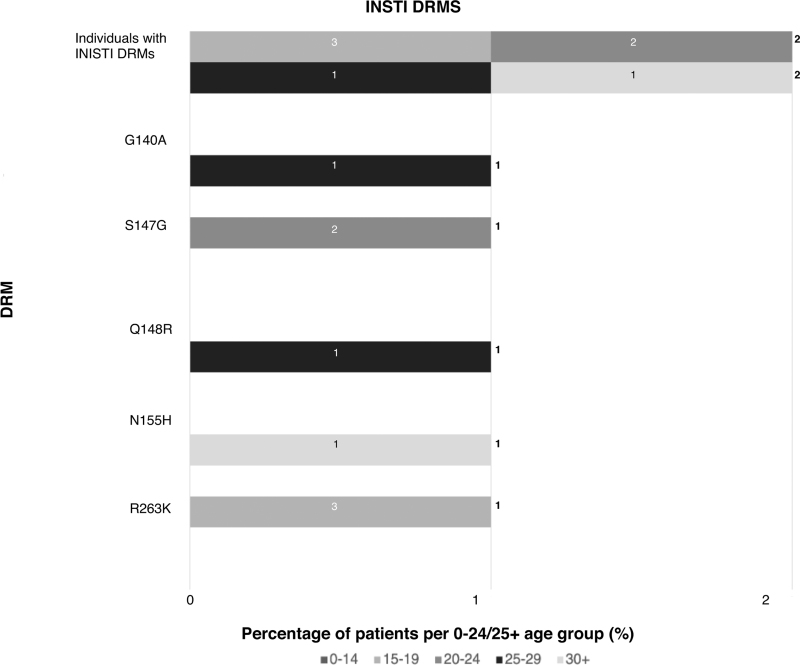
Distribution of age groups for number of resistance classes and antiretroviral therapy class-specific resistance.

The numbers within the bars correspond to the percentage of patients with the mutation per smaller age group (e.g., 0–14, etc.). The numbers outside the bar correspond to the percentage of patients in the larger age group (0–24, 25+) with the mutation. ART, antiretroviral therapy; INSTI, integrase strand transfer inhibitor; NNRTI, nonnucleoside reverse transcriptase inhibitor; NRTI, nucleoside reverse transcriptase inhibitor; PI, protease inhibitor.

**Fig. 2:**
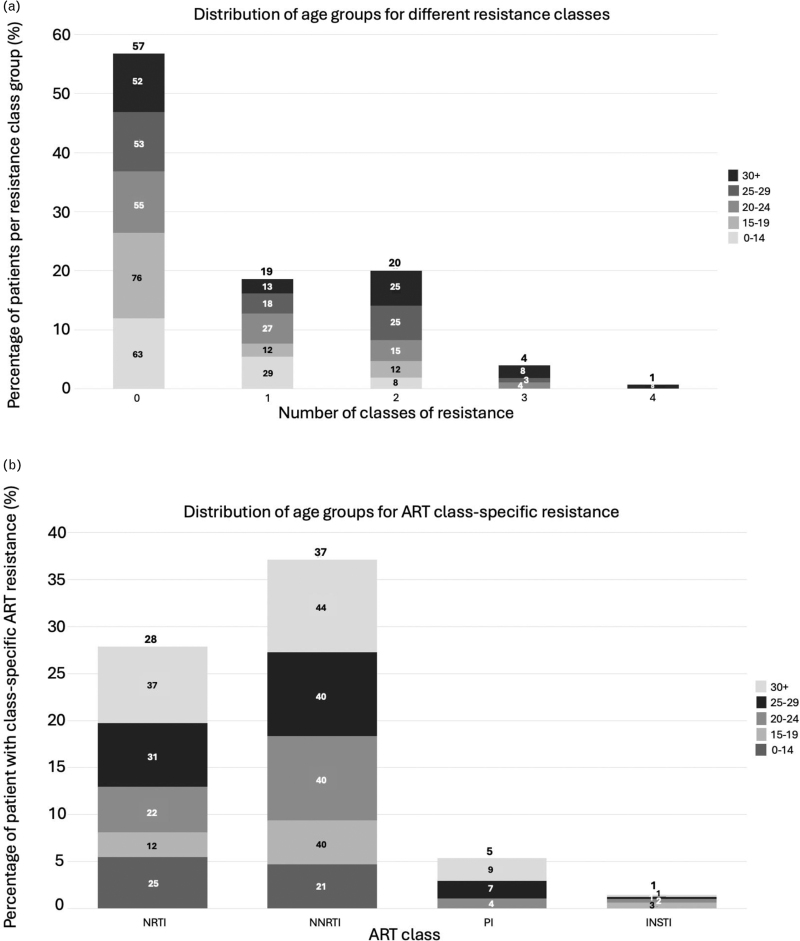
Composite figure of drug resistance mutations according to drug class by frequency and age groups (0–24 and 25+).

Over the last decade in HIC, with better tolerated, higher resistance barrier drugs, multiclass resistance in PaHIV has declined, with triple- and quadruple-class resistance estimated at 6.7% and less than 2%, respectively, aligning with this study's findings (4 and 1%) [4]. The majority (85%) of individuals in this cohort with triple+ class resistance were from the 25+ group, exposed to historic NRTI mono/dual therapy and subsequently to low genetic barrier regimens including unboosted protease inhibitors and NNRTIs (Fig. 2a--d) [7]. Reassuringly, protease and integrase resistance rates were low (5.4 and 1.1%, respectively) and favorable when compared to other HIC cohorts (protease 19–35%, integrase 0–27%; Table 1, Supplementary Table 1) [32–35]. Limited treatment options available for patients with multiclass resistance highlight the need for novel ART classes and modalities including long-acting injectable therapy [7,59].

Refer to Supplementary Table 1 for further details on DRMs of smaller age groups. The numbers within the bars correspond to the percentage of patients with the mutation per smaller age group (e.g., 0–14, etc.). The numbers outside the bar correspond to the percentage of patients in the larger age group (0–24, 25+) with the mutation. DRM, drug resistance mutation; INSTI, integrase strand transfer inhibitor; NNRTI, nonnucleoside reverse transcriptase inhibitor; NRTI, nucleoside reverse transcriptase inhibitor; PI, protease inhibitor.

### Nonnucleoside reverse transcriptase inhibitor resistance and impact on long-acting injectable therapy

Previous virological failure on NNRTI-based regimens may exclude patients from the only currently licenced LA-ART; rilpivirine (LA-RPV), and cabotegravir (LA-CAB) [60,61]. LA-ART CAB/RPV is approved by the FDA from 12 years and at least 35 kg as a suppressed switch option with trials ongoing in younger children (cabotegravir and rilpivirine long-acting injections in young children (CRAYON) Study [NCT05660980]). The More Options for Children and Adolescents (MOCHA) Study [NCT03497676]) enrolled virally suppressed 12–17 year-olds from Botswana, South Africa, Thailand, and USA and demonstrated comparable pharmacokinetic parameters to adults, with high acceptability and tolerability [62,63]. More recently the CARES study showed noninferiority of LA-ART to standard oral ART (96 vs. 97% week 48 endpoint; viral load <50 copies/ml) in a pan African setting in adults with no prior ART failure [64]. However, in contrast, three quarters of our cohort have been exposed to NNRTIs with documented NNRTI-DRMs seen in almost half (Fig. 1   b). The implications of previous NNRTI failure with mutations such as the K103N that do not impact directly on rilpivirine remains uncertain, but our case highlights the need for caution and the potential for low-level archived rilpivirine associated mutations not identified on routine genotypic sequencing [57,65,66]. In LMIC, until the recent roll out of dolutegravir, first line use of NNRTIs was widespread in children with resistance rates approaching 90% in those with virological failure [67]. Currently, NNRTI resistance exceeds 10% worldwide among all individuals with HIV, and without further action an additional 890 000 AIDS-related deaths are predicted by 2030 [68]. Data within perinatal cohorts suggest rates of NNRTI resistance nearer 30%, a reflection of higher rates of virological failure and delayed access to higher genetic barrier regimens for children (Table 1). The development of LA-ART that does not include NNRTIs is urgently required, particularly for youth with unsuppressed viraemia struggling with adherence to daily oral regimens [59,69].

### Emerging integrase strand transfer inhibitor resistance

Within the cohort, INSTIs are now the most common anchor class (*n* = 205, 73%) across all age groups (Table 2, Fig. 2) aligned with WHO guidance [70,71]. Despite their high genetic barrier and relatively recent widespread use, INSTI-DRMs are beginning to emerge, associated with failure on second line regimens with prior NRTI-DRMs [28]. The perinatal Virtual Clinic (https://www.chiva.org.uk/ourworkprof/regional-networks/perinatal-virtual-clinic/) reviews complex cases referred from HIC and LMIC [72]. Although there is potential for selection bias with more referrals for heavily treatment-experienced children and adolescents with access to resistance sequencing, in 2024, integrase resistance was reported in 17/96 (18%) CAPaHIV with virological failure on INSTI-ART and genotypic sequences, and was most frequent in treatment-experienced adolescents from LMIC [29]. A comparable study in Malawi (*n* = 302, age 2–14 years) identified INSTI-DRMs in 17% experiencing persistent virological failure on DTG-ART [20]. In regions with limited viral load monitoring and restricted access to genotyping, potential delays in identifying virological failure may result in a higher risk of acquiring INSTI-DRMs with ongoing viral replication [73]. Univariate statistical analysis in this study found that increased years since ART initiation was significantly associated with the presence of DRMs (Table 3). This challenge is more pronounced in resource-limited settings, where limited options for transition to other anchor classes may hinder the long-term sustainability of INSTIs in second-line therapy, highlighting the ongoing need for access to boosted protease inhibitors in all settings, including palatable formulations for younger children [28].

### Risk factors associated with the acquisition of drug resistance mutations

Historic exposure to mono/dual therapy with NRTIs was much higher in the 25+ group compared to the 0–24 group (17 vs. 1%) with NRTI-DRMs also more common (34 vs. 20%). This likely reflects the fact that these are two different ART eras; those aged 25 years and older typically survived early childhood without access to ART and subsequently commenced low genetic barrier regimens based around NNRTIs. A proportion of this age group also experienced mono-dual NRTI in the latter half of the 1990 s prior to the availability of triple therapy. Both univariate and multivariate analysis found prior mono/dual NRTI therapy significantly increased the life time risk of acquisition of DRMs (*P* = 0.000, 0.029) (Table 3). Exposure to more anchor classes was also associated with an increased risk of DRMs in this population. This may reflect challenges with tolerance or adherence, and also time on ART. It is however important to note that the presence of DRM may lead to class switching so anchor class exposure could be a consequence of, as well as a predictor for, DRM.

Univariate analysis found an association of DRMs with increasing age (*P* = 0.052) and total years since ART initiation (*P* = 0.000). The risk of acquiring DRMs increases with longer ART duration if virological suppression is not maintained [7]. The median duration since ART initiation was 11 years (IQR 6,17) for those aged 0–24 years and 20 (IQR 16,24) for those aged 25+ years (Table 2). Factors such as pill fatigue and the developmental period of adolescence are associated with poorer adherence to therapy and health management for many chronic diseases of childhood [74,75]. For those living with HIV, adherence is further complicated by HIV-associated stigma and the frequent need to hide medication from friends and family [76]. Length of ART exposure and previous inadequate formulations in early childhood mean that rates of triple-class resistance are higher in young adults with PaHIV than those who acquired HIV horizontally, with an increased potential risk of transmission of resistant variants to both partners and offspring [1]. The prioritization of unsuppressed adolescents and young adults for programmatic implementation and research on long-acting therapeutic strategies is essential if the goal of ending the HIV epidemic is to be realized [52]. To reduce the access gap for newer therapies, there are increasingly strong arguments for the routine inclusion of adolescents age 10–17 years weighing at least 35 kg in phase 3 clinical trials with adults [59]. Expert patient involvement is essential, providing unique insights and lived patient experience, particularly from young people who have lived with HIV their entire lives [77].

### Limitations

Limitations include incomplete datasets, including missing resistance assays, pre-ART start data, and treatment history, especially for patients who transferred care on suppressive ART. For those on suppressive therapy, routine HIV RNA genotyping is not possible, preventing identification of prior archived DRMs associated with virological failure on previous ART regimens in other settings. This may have particular implications for those migrating from LMIC settings suppressed on DTG-ART wishing to switch to LA-CAB/RPV or oral dual therapy where a history of prior NNRTI failure is unknown. The clinical utilization of proviral DNA sequencing in this setting remains unknown [7,78]. The mechanisms behind the acquisition of DRMs are multifaceted and complex and whilst this study provides some insight into potential risk factors, inferences are limited by samples size and observational nature of the data. Anchor class exposure has been included in the model as an ordinal variable but it is important to caveat that each drug class is not equal. Assessment of the risk of DRM with individual drug classes is beyond the scope of these data, however we have included sensitivity analyses in the supplementary materials to explore the relationship between anchor drug class and predictors of resistance.

The study's strengths include the duration of follow-up, at least a decade longer than published data in PaHIV. Indeed, life course data on DRM acquisition in adults with PaHIV who have transitioned to adult care are extremely sparse.

## Conclusion

The study highlights the complex landscape of acquired HIV drug resistance in the context of perinatal HIV and lifelong exposure to HIV and increasingly ART. The challenges of ART adherence in infancy, childhood and in adolescence underscore the need for continued research and innovation in LA-ART formulations and patient-centered approaches for these populations. Future efforts should focus on inclusive treatment strategies involving patients in shaping more accessible ART regimens to mitigate development of drug resistance and improve outcomes for those living with HIV, prioritizing vulnerable groups including children and adolescents.

## Acknowledgements

### Conflicts of interest

There are no conflicts of interest.

## Supplementary Material
