# Supplementary material for: The cumulative prevalence of HIV-1 drug resistance in perinatal HIV
Source: AIDS. 2025 Apr 15;39(9):1161–77. doi: 10.1097/QAD.0000000000004202 (PMC12237116; doi:10.1097/QAD.0000000000004202)
Supplement: Supplementary file 1 [file aids-39-1161-s001.docx]

| SUPPLEMENTARY TABLES/FIGURES  Supplementary 1: Drug resistance mutations | | | | | | | | | | | | | | | | |  |
| --- | --- | --- | --- | --- | --- | --- | --- | --- | --- | --- | --- | --- | --- | --- | --- | --- | --- |
|  | |  | **Age group / years** | | | | | | | | | | |  | |  |  |
|  | |  | **0-14** | | | **15-19** | | **20-24** | | **25-29** | | **30+** | | **0-24** | | **25+** |  |
|  | | n (%) | 24 (9) | | | 34 (12) | | 55 (20) | | 88 (31) | | 79 (28) | | 113 (40) | | 167 (60) |  |
| NRTI | |  |  | | |  | |  | |  | |  | |  | |  |  |
| Mutation | |  |  | | |  | |  | |  | |  | |  | |  |  |
| M184V/I | |  | 6 | | | 3 | | 12 | | 19 | | 20 | | 21 | | 39 |  |
|  | | n (%) | 6 (25) | | | 3 (9) | | 12 (22) | | 19 (22) | | 20 (25) | | 21 (19) | | 39 (23) |  |
| TAMs | |  |  | | |  | |  | |  | |  | |  | |  |  |
| M41L | | n (%) | 0 | | | 1 (3) | | 1 (2) | | 4 (5) | | 9 (11) | | 2 (2) | | 13 (8) |  |
| D67N | | n (%) | 0 | | | 0 | | 3 (5) | | 9 (10) | | 13 (16) | | 3 (3) | | 22 (13) |  |
| K70R | | n (%) | 1 (4) | | | 0 | | 1 (2) | | 6 (7) | | 9 (11) | | 2 (2) | | 15 (9) |  |
| L210W | | n (%) | 0 | | | 0 | | 0 | | 2 (2) | | 6 (8) | | 0 | | 8 (5) |  |
| T215C/D/F/N/S/T/V/Y | | n (%) | 0 | | | 0 | | 2 (4) | | 6 (7) | | 14 (18) | | 2 (2) | | 20 (12) |  |
| K219E/N/R/Q | | n (%) | 1 (4) | | | 0 | | 1 (2) | | 7 (8) | | 13 (16) | | 2 (2) | | 20 (12) |  |
| Others | |  |  | | |  | |  | |  | |  | |  | |  |  |
| E44A/D | | n (%) | 0 | | | 0 | | 0 | | 2 (2) | | 2 (3) | | 0 | | 4 (2) |  |
| A62V | | n (%) | 0 | | | 0 | | 0 | | 1 (1) | | 1 (1) | | 0 | | 2 (1) |  |
| K65N/R | | n (%) | 1 (4) | | | 0 | | 1 (2) | | 3 (3) | | 3 (4) | | 2 (2) | | 6 (4) |  |
| D67G/H | | n (%) | 0 | | | 0 | | 0 | | 2 (2) | | 2 (3) | | 0 | | 4 (2) |  |
| T69D | | n (%) | 0 | | | 0 | | 0 | | 1 (1) | | 0 | | 0 | | 1 (1) |  |
| K70E/T | | n (%) | 0 | | | 0 | | 0 | | 1 (1) | | 2 (3) | | 0 | | 3 (2) |  |
| L74I/V | | n (%) | 0 | | | 1 (3) | | 1 (2) | | 6 (7) | | 5 (6) | | 2 (2) | | 11 (7) |  |
| V75I/M/T | | n (%) | 0 | | | 0 | | 1 (2) | | 1 (1) | | 4 (5) | | 1 (1) | | 5 (3) |  |
| Y115F | | n (%) | 0 | | | 1 (3) | | 0 | | 4 (5) | | 0 | | 1 (1) | | 4 (2) |  |
| F77L | | n (%) | 0 | | | 0 | | 0 | | 0 | | 1 (1) | | 0 | | 1 (1) |  |
| F116Y | | n (%) | 0 | | | 0 | | 0 | | 1 (1) | | 0 | | 0 | | 1 (1) |  |
| NNRTI |  | | |  |  | |  | |  | |  | |  | |  | |  |
| Mutation | |  |  | | |  | |  | |  | |  | |  | |  |  |
| A98G | | n (%) | 0 | | | 0 | | 2 (4) | | 0 | | 5 (6) | | 2 (2) | | 5 (3) |  |
| L100I | | n (%) | 0 | | | 0 | | 0 | | 1 (1) | | 0 | | 0 | | 1 (1) |  |
| K101E/H | | n (%) | 0 | | | 0 | | 2 (4) | | 5 (6) | | 3 (4) | | 2 (2) | | 8 (5) |  |
| K103N/S | | n (%) | 4 (17) **** | | | 2 (6) | | 6 (11) | | 15 (17) | | 23 (29) | | 12 (11) | | 38 (23) |  |
| V106A/I/M | | n (%) | 0 | | | 2 (6) | | 3 (5) | | 3 (3) | | 1 (1) | | 5 (4) | | 4 (2) |  |
| V108I | | n (%) | 0 | | | 0 | | 3 (5) | | 5 (6) | | 3 (4) | | 3 (3) | | 8 (5) |  |
| E138G/K | | n (%) | 0 | | | 0 | | 1 (2) | | 1 (1) | | 2 (3) | | 1 (1) | | 3 (2) |  |
| V179E/L/T | | n (%) | 0 | | | 1 (3) | | 0 | | 1 (1) | | 1 (1) | | 1 (1) | | 2 (1) |  |
| Y181C/V | | n (%) | 1 (4) | | | 2 (6) | | 12 (22) | | 16 (18) | | 18 (23) | | 15 (13) | | 34 (20) |  |
| Y188C/H/L | | n (%) | 0 | | | 1 (3) | | 3 (5) | | 4 (5) | | 0 | | 4 (4) | | 4 (2) |  |
| G190A/E/S | | n (%) | 0 | | | 1 (3) | | 4 (7) | | 8 (9) | | 7 (9) | | 5 (4) | | 15 (9) |  |
| H221Y | | n (%) | 1 (4) | | | 0 | | 1 (2) | | 2 (2) | | 2 (3) | | 2 (2) | | 4 (2) |  |
| P225H | | n (%) | 0 | | | 0 | | 0 | | 1 (1) | | 2 (3) | | 0 | | 3 (2) |  |
| F227L | | n (%) | 0 | | | 1 (3) | | 1 (2) | | 1 (1) | | 1 (1) | | 2 (2) | | 2 (1) |  |
| M230I | | n (%) | 0 | | | 0 | | 1 (2) | | 0 | | 0 | | 1 (1) | | 0 |  |
| K238T | | n (%) | 0 | | | 0 | | 0 | | 1 (1) | | 1 (1) | | 0 | | 2 (1) |  |
| PI | | | | | | | | | | | | | | | | |  |
| Mutation | |  |  | | |  | |  | |  | |  | |  | |  |  |
| D30N | | n (%) | 0 | | | 0 | | 1 (2) | | 0 | | 1 (1) | | 1 (1) | | 1 (1) |  |
| V32I | | n (%) | 0 | | | 0 | | 0 | | 0 | | 1 (1) | | 0 | | 1 (1) |  |
| M46I | | n (%) | 0 | | | 0 | | 1 (2) | | 2 (2) | | 3 (4) | | 1 (1) | | 5 (3) |  |
| I54L/V | | n (%) | 0 | | | 0 | | 1 (2) | | 1 (1) | | 0 | | 1 (1) | | 1 (1) |  |
| V82A/S | | n (%) | 0 | | | 0 | | 0 | | 2 (2) | | 1 (1) | | 0 | | 3 (2) |  |
| I84V | | n (%) | 0 | | | 0 | | 0 | | 0 | | 1 (1) | | 0 | | 1 (1) |  |
| N88G/S | | n (%) | 0 | | | 0 | | 0 | | 0 | | 2 (3) | | 0 | | 2 (1) |  |
| L90M | | n (%) | 0 | | | 0 | | 0 | | 3 (3) | | 4 (5) | | 0 | | 7 (4) |  |
| INSTI | | | | | | | | | | | | | | | | |  |
| Mutation | |  | 0 | | | 0 | | 0 | | 1 | | 0 | | 0 | | 1 |  |
| G140A | | n (%) | 0 | | | 0 | | 0 | | 1 (1) | | 0 | | 0 | | 1 (1) |  |
| S147SG | | n (%) | 0 | | | 0 | | 1 (2) | | 0 | | 0 | | 1 (1) | | 0 |  |
| Q148R | | n (%) | 0 | | | 0 | | 0 | | 1 (1) | | 0 | | 0 | | 1 (1) |  |
| N155H | | n (%) | 0 | | | 0 | | 0 | | 0 | | 1 | | 0 | | 1 (1) |  |
| R263K | | n (%) | 0 | | | 1 (3) | | 0 | | 0 | | 0 | | 1 (1) | | 0 |  |

Abbreviations: NRTI – nucleoside-reverse transcriptase inhibitor. TAMs – thymidine analogues mutations. NNRTI – non-nucleoside reverse transcriptase inhibitor. PI – protease inhibitor. INSTI – integrase strand transfer inhibitor.

**Supplementary Table 2: Comparison of demographic data between age groups**

|  | **Age Group/years** | |  |
| --- | --- | --- | --- |
|  | **0-24** n (%) | **25+** n (%) | **p-value*** |
|  | 113 (40) | 167 (60) |  |
| Female | 62 (55) | 98 (59) | 0.541 |
| Black African | 90 (80) | 145 (87) | 0.135 |
| Country of birth - Abroad | 54 (48) | 108 (65) | **0.007** |
| Clade |  |  |  |
| C | 42 (51) | 45 (34) | **0.016** |
| B | 5 (6) | 13 (10) | **0.043** |
| Other | 36 (43) | 76 (57) |  |
| Prior CDC-C Status | 53 (47) | 98 (59) | 0.067 |
| Latest viral load (<200 c/ml) | 105 (93) | 137 (88) | 0.225 |
| Latest viral load (>1000 c/ml) | 3 (3) | 16 (10) | **0.028** |
| Nadir CD4 Count ≥350 cells/µl | \| 71 (65) \|  \| \| --- \| --- \| | 22 (16) | **<0.0001** |
| Past INSTI exposure | 100 (89) | 141 (84) | 0.382 |
| Past NNRTI exposure | 77 (68) | 142 (85) | **0.001** |
| Past boosted PI exposure | 62 (55) | 129 (77) | **0.0001** |
| Past unboosted PI exposure | 3 (3) | 30 (18) | **<0.0001** |
| Past Other ** | 2 (2) | 11 (7) | 0.082 |
| Past mono/dual NRTI therapy | 1 (1) | 28 (17) | **<0.0001** |
| Current INSTI | 95 (84) | 110 (66) | **0.0009** |
| Current NNRTI | 9 (8) | 14 (8) | >0.999 |
| Current Boosted PI | 9 (8) | 31 (19) | **0.015** |
| Current Boosted PI and INSTI | 1 (1) | 9 (5) | 0.053 |
| Current NNRTI and INSTI  (LA-ART) | 0 | 3 (2) | 0.275 |
| Current NIL treatment | 1 (1) | 0 | 0.404 |
| Number of classes of resistance |  |  |  |
| 0 | 71 (63) | 88 (53) | 0.110 |
| 1 | 26 (23) | 26 (16) | 0.121 |
| 2 | 14 (12) | 42 (25) | **0.010** |
| 3 | 2 (2) | 9 (5) | 0.209 |
| 4 | 0 | 2 (1) | 0.517 |
| Multi-Class Resistance (2+) | 16 (14) | 53 (32) | **0.001** |
| Any NRTI mutation | 22 (20) | 56 (34) | **0.010** |
| M184V/I | 21 (19) | 39 (23) | 0.375 |
| TAMs | 6 (5) | 6 (4) | 0.554 |
| Other | 5 (4) | 27 (16) | **0.002** |
| Any NNRTI mutation | 34 (30) | 70 (42) | 0.058 |
| Any PI mutation | 2 (2) | 13 (8) | **0.031** |
| Any INSTI mutation | 2 (2) | 2 (1) | >0.999 |

Abbreviations: CDC-C - The Centers for Disease Control and Prevention C status. INSTI – integrase strand transfer inhibitor. NNRTI – non-nucleoside reverse transcriptase inhibitor. PI – protease inhibitor. NRTI – nucleoside-reverse transcriptase inhibitor. LA-ART – long acting antiretroviral therapy. TAMs – thymidine analogues mutations.

p<0.05 was considered significant. All significant p-values were bolded.

*To compare categorical variables between groups, Chi-square or Fisher’s exact tests were used depending on number of variables.

**Supplementary Table 3: Type of ART resistance according to age groups**

|  |  | **0-14** | **15-19** | **20-24** | **25-29** | **30+** | **0-24** | **25+** |
| --- | --- | --- | --- | --- | --- | --- | --- | --- |
|  | **n** | **24** | **34** | **55** | **88** | **79** | **113** | **167** |
| **Only NRTI** | n (%) | 4 (17) | 0 | 2 (4) | 3 (3) | 1 | 6 (5) | 4 (2) |
| **Only NNRTI** | n (%) | 3 (13) | 3 (9) | 12 (22) | 13 (15) | 8 (10) | 18 (16) | 21 (13) |
| **Only PI** | n (%) | 0 | 0 | 0 | 0 | 1 (1) | 0 | 1 (1) |
| **Only INSTI** | n (%) | 0 | 1 (3) | 1 (2) | 0 | 0 | 2 (2) | 0 |
| **NRTI + NNRTI** | n (%) | 2 (8) | 4 (12) | 8 (15) | 19 (22) | 19 (24) | 14 (12) | 38 (23) |
| **NRTI + PI** | n (%) | 0 | 0 | 0 | 3 (3) | 1 (1) | 0 | 4 (2) |
| **NRTI + NNRTI + PI** | n (%) | 0 | 0 | 2 (4) | 3 (3) | 6 (8) | 2 (2) | 9 (5) |
| **NRTI + NNRTI + PI + INSTI** | n (%) | 0 | 0 | 0 | 0 | 2 (3) | 0 | 2 (1) |

**Supplementary Table 3: Type of ART resistance according to age groups**

Abbreviations: ART – antiretroviral therapy. NRTI – nucleoside reverse transcriptase inhibitor. NNRTI – non-nucleoside reverse transcriptase inhibitor. PI – protease inhibitor. INSTI – integrase strand transfer inhibitor.

For the first four categories (NRTI, NNRTI, PI, and INSTI), individuals only had single-class resistance to the corresponding ART class. For NRTI+NNRTI and NRTI+PI, individuals had dual-class resistance to these ART anchor classes. Finally, individuals in the NRTI+NNRTI+PI group had triple-class resistance and NRTI+NNRTI+PI+INSTI had quadruple-class resistance.

**Multivariable model methods**

Univariable analysis was conducted between DRMs (outcome variable) and all potential exposure variables in turn. All exposure variables where p <0.1 were then tested for inclusion in the final model. The variable with the largest association with the outcome on univariable analysis was the first selected for inclusion in the multivariable model. Regression analysis was conducted using this model and each subsequent exposure variable in turn working sequentially from largest to smallest association. Nested models were compared using likelihood ratio tests to see if newly added variables improved model fit. The variable which most improved model fit from this regression analysis (smallest p value on likelihood ratio test) became the newly selected variable and included in the model. The cycle continued until no more exposure variables were able to be included. If a variable improved the model it was included, and retained in the model even if it subsequently lost significance. ^[1]^

Age was included in the model a-priori because it was felt by the authors to be potentially important to the outcome variable. Gender was not adjusted for a-priori as the likelihood of a significant association was considered to be much lower, and to reduce unnecessary variables in the model. It was tested in the multivariable analysis but not retained in the model. CD4 nadir and latest viral load were not included in the model because of a-priori concerns regarding association with the outcome variable (DRMs).

Missing data were assumed to be missing at random. Models were fit on observations with complete sets of data.

Collinearity diagnostics were performed on the model with results In Supplementary Table 4.

**Supplementary Table 4: Collinearity diagnostics**

**Supplementary Table 4: Collinearity diagnostics**

Abbreviations: VIF – variance inflation factor. ART – antiretroviral therapy. NRTI – nucleoside reverse transcriptase inhibitor. CDC-C - The Centers for Disease Control and Prevention C status.

|  | **VIF** | **Tolerance** |
| --- | --- | --- |
| Years since ART initiation | 1.45 | 0.69 |
| Anchor class exposure | 1.14 | *0.88* |
| Prior mono/dual NRTI therapy | 1.12 | 0.89 |
| Age in years | 1.29 | 0.78 |
| Prior CDC-C diagnosis | 1.09 | 0.92 |

Variables were included in multivariable analysis based on apriori knowledge of potential associations with DRM.

The following variables were included:

Age in years

Ethnicity

Gender

Country of birth

Weight

Height

Pretreatment viral load

Pre-treatment CD4 count

Anchor class exposure (number)

Hepatitis B infection

Hepatitis C infection

Prior The Centers for Disease Control and Prevention C status (CDC-C) diagnosis

Years since ART initiation

Prior mono/dual NRTI therapy exposure

HIV subtype clade

**Sensitivity analysis**

Several sensitivity analyses were run.

First the model was run on the cohort stratified by exposure to key drug classes. These were integrase strand transfer inhibitor (INSTI) exposed/unexposed, non-nucleoside reverse transcriptase inhibitor (NNRTI) exposed/unexposed, boosted protease inhibitor (PI) exposed and unexposed and unboosted PI exposed and unexposed.

Second the model was re-run without including anchor class exposure as a variable to evaluate the effect this had on the model. This was done because number of anchor classes exposed to could be both a risk factor and a consequence of drug resistance mutations (DRMs).

Thirdly the model was run with the cohort stratified by age into <25 and >=25.

The results of these are in supplementary tables 5-12.

1. Predictors of DRMs for individuals exposed to different drug classes

**INSTI Exposed: N= 241**

**Supplementary Table 5: Predictors of DRMs for individuals exposed to INSTIs**

|  | **OR (95% CI)** | **P value** |
| --- | --- | --- |
| Age in years | 0.85 (0.65, 1.12) | 0.257 |
| Years since ART initiation | 1.46 (0.89, 2.39) | 0.129 |
| Anchor class exposure | 7.39 (3.79, 14.39) | <0.001 |
| Prior mono/dual NRTI therapy exposure | 4.07 (1.05, 15.74) | 0.042 |
| Prior CDC-C diagnosis | 1.25 (0.67, 2.30) | 0.480 |

**Supplementary Table 5: Predictors of DRMs for individuals exposed to INSTIs**

Abbreviations: DRMs – drug resistance mutations. INSTIs – integrase strand transfer inhibitors. OR – odds ratio. CI – confidence interval. ART – antiretroviral therapy. NRTI - nucleoside reverse transcriptase inhibitor. CDC-C - The Centers for Disease Control and Prevention C status.

**INSTI unexposed: N=39**

Model unable to converge due to collinearity

**NNRTI exposed: N=219**

**Supplementary Table 6: Predictors of DRMs for individuals exposed to NNRTIs**

|  | **OR (95% CI)** | **P value** |
| --- | --- | --- |
| Age in years | 0.95 (0.72, 1.29) | 0.774 |
| Years since ART initiation | 1.42 (0.83, 2.40) | 0.198 |
| Anchor class exposure | 6.91 (3.56, 13.41) | <0.001 |
| Prior mono/dual NRTI therapy exposure | 3.35 (1.05, 10.7) | 0.041 |
| Prior CDC-C diagnosis | 1.54 (0.82, 2.93) | 0.179 |

**Supplementary Table 6: Predictors of DRMs for individuals exposed to NNRTIs**

Abbreviations: DRMs – drug resistance mutations. NNRTIs – non-nucleoside reverse transcriptase inhibitors. OR – odds ratio. CI – confidence interval. ART – antiretroviral therapy. NRTI - nucleoside reverse transcriptase inhibitor. CDC-C - The Centers for Disease Control and Prevention C status.

**NNRTI unexposed: N=60**

Model unable to converge due to collinearity

**Boosted PI exposed: N=191**

**Supplementary Table 7: Predictors of DRMs for individuals exposed to boosted PIs**

|  | **OR (95% CI)** | **P value** |
| --- | --- | --- |
| Age in years | 0.96 (0.71, 1.30) | 0.783 |
| Years since ART initiation | 1.16 (0.66, 2.02) | 0.600 |
| Anchor class exposure | 4.06 (2.02, 8.13) | <0.001 |
| Prior mono/dual NRTI therapy exposure | 6.09 (1.29, 28.7) | 0.022 |
| Prior CDC-C diagnosis | 1.45 (0.74, 2.81) | 0.278 |

**Supplementary Table 7: Predictors of DRMs for individuals exposed to boosted PIs**

Abbreviations: DRMs – drug resistance mutations. PIs – protease inhibitors. OR – odds ratio. CI – confidence interval. ART – antiretroviral therapy. NRTI - nucleoside reverse transcriptase inhibitor. CDC-C - The Centers for Disease Control and Prevention C status.

**Boosted PI unexposed: N=81**

Model unable to converge due to collinearity

**Unboosted PI exposed: N=33**

**Supplementary Table 8: Predictors of DRMs for individuals exposed to unboosted PIs**

|  | **OR (95% CI)** | **P value** |
| --- | --- | --- |
| Age in years | 1.35 (0.37, 4.92) | 0.654 |
| Years since ART initiation | 2.24 (0.50, 10.08) | 0.291 |
| Anchor class exposure | 2.84 (0.42, 19.32) | 0.287 |
| Prior mono/dual NRTI therapy exposure | 2.73 (0.48, 28.57) | 0.287 |
| Prior CDC-C diagnosis | 3.00 (0.38, 23.53) | 0.296 |

**Supplementary Table 8: Predictors of DRMs for individuals exposed to unboosted PIs**

Abbreviations: DRMs – drug resistance mutations. PIs – protease inhibitors. OR – odds ratio. CI – confidence interval. ART – antiretroviral therapy. NRTI - nucleoside reverse transcriptase inhibitor. CDC-C - The Centers for Disease Control and Prevention C status.

**Unboosted PI unexposed: N=247**

**Supplementary Table 9: Predictors of DRMs for individuals unexposed to unboosted PIs**

|  | **OR (95% CI)** | **P value** |
| --- | --- | --- |
| Age in years | 0.89 (0.69, 1.14) | 0.374 |
| Years since ART initiation | 1.42 (0.90, 2.21) | 0.132 |
| Anchor class exposure | 6.34 (3.60, 11.19) | 0.000 |
| Prior mono/dual NRTI therapy exposure | 3.51 (1.13, 10.91) | 0.029 |
| Prior CDC-C diagnosis | 1.64 (0.93, 2.90) | 0.085 |

**Supplementary Table 9: Predictors of DRMs for individuals unexposed to unboosted PIs**

Abbreviations: DRMs – drug resistance mutations. PIs – protease inhibitors. OR – odds ratio. CI – confidence interval. ART – antiretroviral therapy. NRTI - nucleoside reverse transcriptase inhibitor. CDC-C - The Centers for Disease Control and Prevention C status.

1. Anchor class exposure not included in model

**Supplementary Table 10: Anchor class exposure not included in model N=280**

|  | **OR (95% CI)** | **P value** |
| --- | --- | --- |
| Age in years | 0.96 (0.77, 1.21) | 0.744 |
| Years since ART initiation | 1.69 (1.13, 2.53.27) | 0.010 |
| Prior mono/dual NRTI therapy exposure | 4.20 (1.47, 12.05) | 0.008 |
| Prior CDC-C diagnosis | 1.83 (1.10, 3.09) | 0.021 |

**Supplementary Table 10: Anchor class exposure**

Abbreviations: OR – odds ratio. CI – confidence interval. ART – antiretroviral therapy. NRTI - nucleoside reverse transcriptase inhibitor. CDC-C - The Centers for Disease Control and Prevention C status.

1. Risk factors for DRMs stratified by age

**Supplementary Table 11: Risk factors for DRMs for those aged >24 years N=167**

|  | OR (95% CI) | P value |
| --- | --- | --- |
| Age | 0.81 (0.38, 1.75) | 0.595 |
| Years since ART initiation | 1.83 (0.93, 3.60) | 0.081 |
| Anchor Class Exposure | 6.93 (3.10, 15.60) | 0.000 |
| Prior mono/dual NRTI therapy exposure | 4.66 (1.14, 19.12) | 0.033 |
| Prior CDC-C diagnosis | 3.16 (1.42,7.02) | 0.005 |
| Sex | 0.47 (0.01, 2.94) | 0.212 |

**Supplementary Table 11: Risk factors for DRMs for those aged >24 years**

Abbreviations: DRMs – drug resistance mutations. OR – odds ratio. CI – confidence interval. ART – antiretroviral therapy. NRTI - nucleoside reverse transcriptase inhibitor. CDC-C - The Centers for Disease Control and Prevention C status.

**Supplementary Table 12: Risk factors for DRMs for those aged <25 years N=113**

|  | **OR (95% CI)** | **P value** |
| --- | --- | --- |
| Age | 1.22 (0.70, 2.11) | 0.481 |
| Anchor Class Exposure | 6.60 (2.80, 15.41) | 0.001 |

**Supplementary Table 12: Risk factors for DRMs for those aged <25 years**

Abbreviations: DRMs – drug resistance mutations. OR – odds ratio. CI – confidence interval. ART – antiretroviral therapy.

**References**

1. Bennett A. Rotavirus transmission in the context of reduced vaccine effectiveness in low income countries; 2017. <https://core.ac.uk/download/pdf/161101706.pdf> [accessed 29 December 2024]
